# Inorganic/Inorganic Composites Through Emulsion Templating

**DOI:** 10.1002/adma.202411352

**Published:** 2024-12-20

**Authors:** Tianhui Jiang, Shitong Zhou, Yinglun Hong, Erik Poloni, Eduardo Saiz, Florian Bouville

**Affiliations:** ^1^ Centre for Advanced Structural Ceramics Department of Materials Imperial College London London SW7 2AZ UK

**Keywords:** ceramic composites, emulsion, magnetic templating, porous materials

## Abstract

Inorganic/inorganic composites are found in multiple applications crucial for the energy transition, from nuclear reactors to energy storage devices. Their microstructures dictate their properties from mass transport to fracture resistance. Consequently, there has been a multitude of processes developed to control them, from powder mixing and the use of short or long fibers, to tape casting for laminates up to recent 3D printing. Here, emulsions and slip casting are combined into a simpler, broadly available, inexpensive processing platform that allows for in situ control of composite microstructure while also enabling complex 3D shaping. This study shows that slip casting of emulsions triggers a two‐step solvent removal, opening the possibility for the conformal coating of pores. This process is showcased by producing strong and lightweight alumina scaffolds reinforced by a conformal zirconia coating. In addition, by manipulating magnetically responsive droplets, their distribution can be controlled, allowing for the formation of inorganic fibers inside an inorganic matrix in situ during slip casting. Using this approach, alumina has been reinforced with aligned metallic iron fibers to prepare composites with a work of fracture an order of magnitude higher than the pure ceramics.

## Graphical abstract

The combination of emulsions, slip casting, and inorganic particle loaded suspensions forms a processing platform that allows the controlled fabrication of intricate inorganic/inorganic composites. This new platform can be used for example to produce strong and lightweight porous samples with pores coated by a conformal layer of a second material or tough fiber‐reinforced composites.

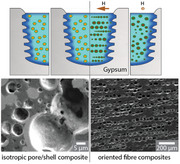

## 1 Introduction

Inorganic/inorganic composites are at the heart of multiple technological materials for the energy transition, from ceramic/ceramic composites for aerospace applications^[^ ^1^, ^2^ ^]^ to fusion nuclear reactors^[^ ^3^ ^]^ or safer and more energy‐dense solid electrolytes for energy storage or fuel cells.^[^ ^4^ ^]^ In all these examples, their microstructure varies in complexity and length scale.^[^ ^5^, ^6^ ^]^

Powder mixing can be used to form a two‐phase composite at the hundred nanometers to a few microns scale, with, for instance, zirconia toughened alumina used in orthopedic applications^[^ ^7^ ^]^ or the next generation of solid electrolytes and electrodes for advanced energy storage.^[^ ^8^ ^]^ Functionally graded microstructure can also be achieved by changing the spatial composition continuously in one direction.^[^ ^8^ ^]^

Going one degree higher in terms of microstructural complexity, 1D structures can be weaved into an inorganic matrix.^[^ ^1^ ^]^ This concept is used in numerous applications, including the well‐known ceramic matrix composites developed for safety‐critical uses, for instance, in high‐temperature reactors, plane engines, or nuclear reactors.^[^ ^3^ ^]^ Fully inorganic long‐ or short‐fiber reinforced composites can also be produced with metallic fiber, for instance, in concrete^[^ ^9^ ^]^ or cermets.^[^ ^9^ ^]^ The fiber diameters range from 10 to 50 µm, and lengths range from 10 µm to millimeters. Multiple processes can be used to fabricate long‐fiber composites, from fiber textile infiltration, short fiber mixing, to more recently 3D printing to fabricate complex architecture in situ.^[^ ^10^, ^11^, ^12^ ^]^

Finally, layered composites have been fabricated using metal or ceramic alternating layers, to reach high mechanical properties by developing crack arresting mechanisms or increasing the performance of capacitors.^[^ ^13^, ^14^, ^15^ ^]^ The processes to obtain layered architectures are mostly based on tape casting, leading to layer thickness varying from 20 µm to 1 millimeters, with some more recent development reaching 10 µm using templating with ice crystals.^[^ ^16^, ^17^, ^18^ ^]^ All these composites are technically critical for numerous applications, and being able to add flexibility and capability in their production could facilitate or open new possibilities.

Emulsions are ubiquitous liquid mixtures that are used in every kitchen and appear in numerous industrial applications.^[^ ^19^ ^]^ They are formed when droplets of an immiscible liquid are stabilized in another using a surfactant.^[^ ^20^, ^21^ ^]^ Droplet sizes can be tuned from half a micron up to a millimeter by manipulating the surfactant amount, stabilization capacity, and mixing energy. The composition can be tuned to modulate the volume fraction of droplets from diluted emulsions up to values close to or even slightly higher than the droplet packing limit ≈60 vol.%^[^ ^22^ ^]^ in conventional emulsions, while high internal phase emulsions can have an oil phase of up to 90 vol.%.^[^ ^23^ ^]^ The applications of emulsions can be found in food products to incorporate fat into water‐based suspensions, in catalysis to form porous structures,^[^ ^24^ ^]^ filtration,^[^ ^25^ ^]^ heat exchange,^[^ ^26^ ^]^ biomedical scaffolds, or up to the fabrication of monodisperse droplets in microfluidic devices for drug encapsulation or alcohol‐free perfumes.^[^ ^27^ ^]^

While the typical emulsion microstructure consists of homogeneously distributed droplets into another fluid matrix, advances in processing can expand our capabilities to control the amount, configuration, and spatial arrangement of the droplets. The addition of magnetically responsive particles within these droplets opens the possibility of controlling their position and concentration using magnetic fields with spatially varying intensity.^[^ ^28^ ^]^ For example, the droplets can form chains whose length and spatial configurations can be adjusted by the magnetic field intensity.^[^ ^26^, ^29^, ^30^ ^]^

Compounding this spatial manipulation, the combination of emulsion templating with particle‐laden droplets, and conventional ceramic processing could provide a new platform to fabricate complex inorganic/inorganic composites with controllable directional microstructures, complex 3D shapes, and tuned compositions. These composites could exhibit a variable anisotropy in their properties and be used in multiple applications from increasing mass transport in fully solid electrodes for energy storage devices to producing catalytic supports, all the way to providing an alternative process to form long fibers inorganic composites in situ for high performance structural applications.

In this study, we use emulsions to implement complex microstructures in inorganic/inorganic composites using particle suspensions in both phases. Because in these conditions the two phases have different densities, we study and modify the rheology of the continuous phase to form a gel using Polyvinyl Alcohol (PVA), critical to avoid creaming or sedimentation issues. These emulsions are then shaped using slip casting, a simple, effective, and industrial‐scale process to make ceramic parts. This process leads to multi‐stage solvent removal that have a strong impact on the final structure, which we study using microscopy and in situ process monitoring. Finally, using this knowledge and process, we demonstrate how this technique can be used first to fabricate strong and lightweight alumina/zirconia composites. In the final example, we show how it can be used to form long metallic iron fiber inside an alumina ceramic matrix using magnetically assisted slip casting^[^ ^31^ ^]^ which shows improved fracture properties.

## 2 Results and Discussion

**Figure 1:**
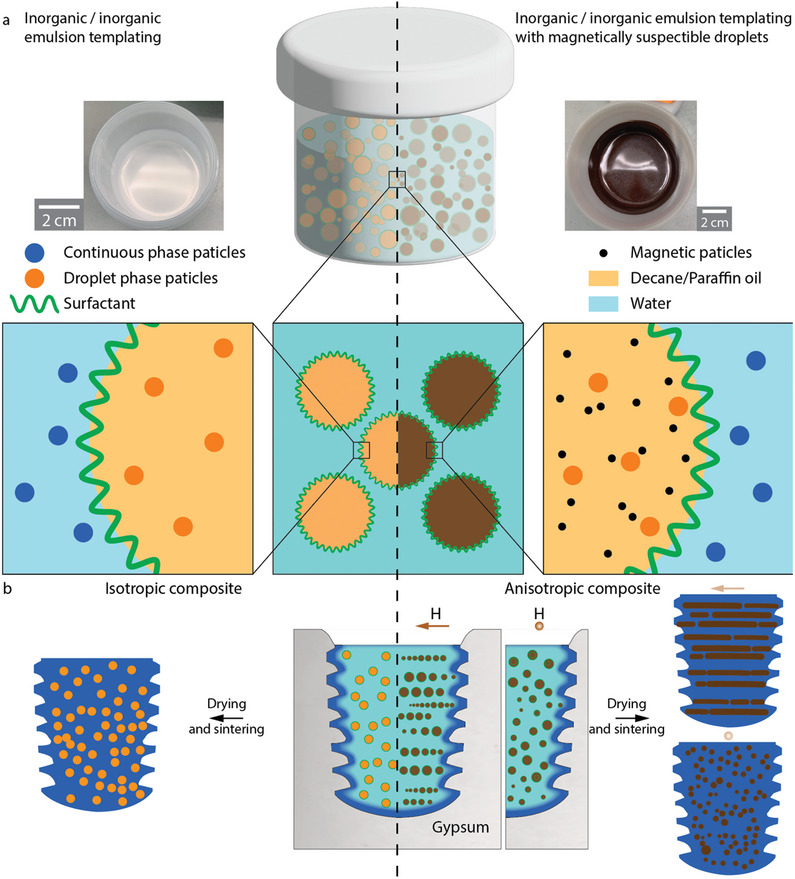
Fabrication of inorganic/inorganic composites through emulsion templating. a) Schematic of the emulsion composition for slip casting. Picture: resulting emulsion after mixing. b) Representation of the slip casting process with and without the magnetic field. The presence of magnetic field induces droplet‐chain formation during the slip casting, leading to anisotropic fiber‐like microstructure.

The microstructure of inorganic/inorganic composites can be controlled by using emulsion templating and the combination of this templating technique with magnetically responsive droplets opens possibilities for the fabrication of more complex architectures. Our strategy to form inorganic/inorganic composites relies on a tweak of the traditional emulsion‐templating formula: instead of using the immiscible liquid in the droplet to form porosity upon drying, the droplet phase contains a stable dispersion of another type of inorganic particles (**Figure** **1**). The initial step is to formulate two stable and well‐dispersed inorganic particles suspensions with two immiscible solvents. A surfactant capable of stabilizing the droplets is then added into the suspension which will form the continuous phase. The two suspensions are mixed with the necessary energy to form droplets, that then coalesce until they are stabilized by the surfactant. We use slip casting to shape the emulsion into complex 3D shapes in an inexpensive and fast way while other, such as drying in a controlled atmosphere, could also be used. Finally, the green body is ready to be densified through isostatic pressing and sintering. The final microstructure is templated by the emulsion, i.e., with a droplet‐shaped assembly of a material dispersed through a matrix (Figure 1a).

The addition of superparamagnetic iron oxide nano particles (SPIONs) into the droplet phase further expands the method as the droplet position and organization in the emulsion can be controlled using a low‐intensity magnetic field (Figure 1a). This enables the formation of droplet chains and potentially graded microstructure.

While this strategy can be used for any composition of materials and couple of immiscible solvents, we choose two examples to demonstrate its potential: alumina/zirconia composites for isotropic emulsion templating and alumina/iron for the anisotropic one. The continuous phase is the same in both cases, with submicrometric alumina (Figure S1, Supporting Information) particles in water dispersed using a poly‐acrylic salt. Different amounts of PVA that will act as the emulsion surfactant are initially dissolved in water before the dispersant and alumina are added. The droplet phases are based on organic solvents, decane for the zirconia‐based suspension (Figure S2, Supporting Information) and a light hydrocarbon oil for the iron oxide‐based one. Both particle types are first coated with a layer of oleic acid to ensure a semi‐steric dispersion in the non‐polar solvents. This coating also increases the hydrophobicity of the particles, leading to contact angle with water of up to 141° and 131° for the zirconia and iron oxide respectively (cf. Figure S3, Supporting Information). The paraffin oil already contains ≈10 vol.% of SPIONs also coated by oleic acid to ensure their dispersion and hydrophobicity. The magnetic field during the slip casting is generated using two rare‐Earth magnets with opposite polarity placed on each side of the slip casting mold, enabling the fabrication of centimeter‐size samples (Figure 1b). The emulsions stability and successful casting is dictated by a delicate balance of the parameters that determine the rheology of the emulsion and can be controlled through its composition.

The stability and the structure of the emulsion can be controlled by both the surfactant and particle concentration. Our emulsions are even more susceptible to settling or creaming than conventional ones as the two liquid phases can present large differences in densities due to the presence of inorganic particles. We choose PVA as a surfactant for simplicity and broad availability and for its capacity to increase the viscosity of the continuous phase. The initial idea was to use this increase in viscosity of the continuous phase to slow a possible settling/creaming of the droplets, however, the role of PVA turned out to be even more important.

**Figure 2:**
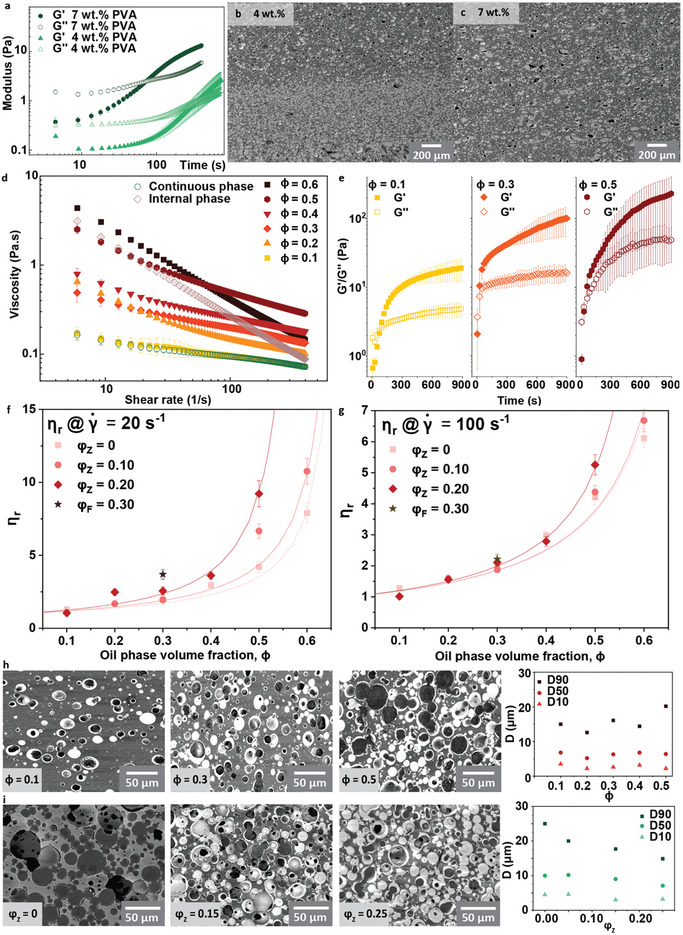
Control of the stability and viscosity of inorganic/inorganic emulsions through rheology modifier. a) Storage G’ and loss G’’ moduli as a function of time for 20 vol.%. alumina slurry in water with 4 and 7 wt.% of PVA. SEM of the microstructure after drying and sintering of water‐alumina/decane‐zirconia emulsion with 4 b) and 7 wt.% c) of PVA. d) Viscosity as a function of shear rate of water‐alumina/decane‐zirconia for different amounts of decane‐zirconia phase ϕ. e) Storage G’ and loss G’’ moduli as a function of time for different amount of decane‐zirconia ϕ. Relative viscosity of emulsions as a function of volume fraction of decane‐zirconia ϕ with different fraction of zirconia φ_*Z*_ or iron in decane φ_*F*_ taken at a shear rate of f) 20 s^−1^ and g) 100 s^−1^. Microstructure of the water‐alumina/decane‐zirconia after slip casting and sintering for h) different amount of decane‐zirconia ϕ at constant zirconia fraction in decane φ_*Z*_ =  0.20 and i) different amount of zirconia in decane φ_*Z*_ at constant ratio of oil phase ϕ  =  0.50 and associate droplet size distribution after sintering.

We used oscillatory rheology to study the evolution of the viscosity of alumina suspensions in water with different concentrations of PVA (**Figure** **2**a). The suspensions exhibit initially a liquid behavior with a loss modulus G’’ higher than the elastic modulus G’. An increase in PVA concentration from 4 to 7 wt.% (with respect to water) increases the elastic modulus of the suspension by almost a factor of five. However, the presence of PVA also triggers the gelation of the suspension over time. The storage moduli (G’) raises above G’’ attesting to a more solid‐like behavior after this point. The gelling time, taken as a first approximation as the crossing time between G’ and G’’, decreases with an increasing amount of PVA, going from more than 5 min to around one when the PVA amount increases from 4 to 7 wt.%.

In the next step, the water‐based suspensions are emulsified with an oil‐based suspension containing 20 vol.% of Zirconia. For clarity, we will use φ as the volume fraction of solid particles in the oil phase and ϕ for the volume fraction of the oil phase, calculated as the volume of oil and volume of particles in it, with respect to the volume of the whole emulsion. The amount of PVA has a strong influence on the final microstructure after casting and sintering. Settling of the zirconia‐containing droplets with 4 wt.% PVA leads to a graded composition from the bottom to the top of the sample (Figure 2b). The emulsions made with 7 wt.% of PVA presented a homogeneous microstructure throughout the sample thickness, with 10 microns diameter droplets of zirconia dispersed within an alumina continuous phase (Figure 2c). We suspect that the higher amount of PVA has two effects: it slows down the movements of the denser zirconia droplets within the alumina water‐based continuous phase while accelerating the gelling that occurs and avoiding further possibilities for the droplet to settle.

Using the 7 wt.% PVA alumina suspension, we can now study the effect of the varying amount of oil‐based zirconia‐suspension on the rheological behavior of the emulsion. Because we want to form dense ceramic/ceramic composites, we study the behavior of emulsions with the highest volume fraction of zirconia and alumina in both phases (20 vol.%). Increasing the volume of oil phase led to an increase in emulsion viscosity (Figure 2d). The shear thinning behavior of the emulsions depends on the oil content. For very low contents, the shear thinning coefficient is similar for the emulsions and the alumina suspensions up to a volume fraction of oil phase ϕ of 0.50, with a viscosity decreasing more rapidly below 20 s^−1^ than above. However, this trend changes for a volume fraction of oil phase of ϕ  =  0.60, with the emulsion becoming more shear thinning (Table S1 and Figure S4, Supporting Information). This behavior is similar to the pure oil suspension, leading us to suspect a phase inversion at this point, with the oil suspension becoming the continuous phase over the water based one, thus dictating the rheological behavior. This phase inversion was confirmed by SEM imaging (Figure S5, Supporting Information) of the resulting composites and the behavior of these emulsions is akin to conventional emulsions.^[^ ^32^ ^]^

We have established that the gelling of the emulsion is key to achieving a homogeneous microstructure in the final composites. The addition of oil phase further shortens the gelation time, from 72 s in the suspensions down to 33 s in the emulsion with the maximum volume fraction of oil (Figure 2e). Increasing the concentration of oil droplets in an emulsion is akin to increase the particles concentration in a conventional ceramic suspension, with the interaction between the droplets dictating more and more of the rheological behavior. The addition of more oil increases the viscosity of the emulsion following a power law, with the viscosity increasing faster the closer the volume fraction of oil‐phase gets to the droplet packing limit. This behavior can be described using the Krieger‐Dougherty model traditionally applied to suspensions with solid particles dispersed in a solvent. The fitting enables the calculation of the packing limit, ϕ_max_:

(1) $$\eta_{r}=\frac{\eta_{Emulsion}}{\eta_{Continuous\;Phase}}={\left(1-\frac{\phi }{\phi_{max}}\right)}^{-\left[\eta \right]\phi_{max}}$$

With [η] being the intrinsic viscosity of the suspension by analogy with the formula developed by Einstein for dilute systems.^[^ ^33^ ^]^ With an oil‐phase containing the maximum volume fraction of zirconia of φ_*Z*_ = 0.20, this packing limit is found to be ϕ_max_ =  0.60 at a low shear rate. The packing limit increases when the amount of zirconia in the droplet is decreased, with a packing limit of ϕ_max_ =  0.65 reached for pure oil with no zirconia. We hypothesized that increasing the amount of zirconia in the droplets increases the viscosity of the suspension within the droplet, leading to more agglomeration and lower packing. This trend is confirmed as the packing fraction extracted from the viscosity at higher shear rate becomes similar for all volume fractions of zirconia in the oil. Finally, these plots allow us to establish the range within which the viscosity of the emulsion is low and thus castable, but also in which the droplets can be manipulated with a magnetic field. For all emulsions, including the ones made with the iron oxide (Figure S6, Supporting Information) and containing SPIONs, the viscosity remains low below a volume fraction of ϕ  =  0.4 of oil (Figure 2f,g).

The microstructure of the emulsion‐templated composites can be characterized using SEM. First, we vary the amount of oil‐phase at a constant φ_*Z*_ =  0.20 volume fraction of zirconia (Figure 2h). The zirconia droplets are hollow, which we assume is due to the presence of solvent in the droplets leading to the formation of porosity, but this will be described in more detail later in the paper. A clear separation is visible on the SEM images between the droplets and continuous phase, with no alumina particles visible in the zirconia shells and vice‐versa (see Figures 2h,i and S7, Supporting Information). It is possible to see droplets partially coalesced in these images (see Figure S7, Supporting Information for a close‐up view). The presence of windows could arise from arrested coalescence^[^ ^34^ ^]^ or shear forces^[^ ^35^ ^]^ during mixing. The reasons behind the porosity and non‐mixing of the two particles populations will be explained in more detail later in the paper. The droplet size distribution, measured using image analysis, remains constant with increasing oil‐phase and varies between D_10_  ≈   3 µm to D_90_  ≈   15 µm until ϕ > 0.4, where the distribution of droplet size broadens in the large pore size, with D90 reaching 20 µm. This broadening of the distribution is also accompanied by an increase in droplet partial coalescence (Figure 2i). This constant distribution for ϕ < 0.4 reflects the stability obtained by the high PVA amount and gelling of the suspensions.

The microstructures of the emulsions obtained by varying the amount of zirconia at a constant volume fraction of oil‐phase of ϕ  =  0.50 are presented in Figure 2i along with the droplets’ size distributions. The addition of zirconia to the oil phase decreases the width of the droplet size distribution, with a D_90_ decreasing from 25 to 17 µm when the zirconia content increases from 0 to 0.25, while D_10_ and D_50_ stay relatively constant. This decrease points again toward a surface stabilizing effect of the zirconia, even if more study would be necessary to study this quantitatively.

By tuning the amount of PVA, we obtained stable emulsions that template the microstructure of ceramic/ceramic composites. The pores resulting from the droplets are all hollow, which can be directly linked with the behavior of both solvents during slip casting.

The formation of hollow zirconia shells in the microstructure of emulsion‐templated composites can be explained by a sequential two step‐slip casting process. All the emulsion‐templated composites exhibit an internal porosity regardless of the composition. While some porosity could be expected from the removal of the solvent from the droplets as the powder in the oil forms a thin shell covering the spherical pores. This observation as well as our curiosity as to how two immiscible solvents can be extracted from an assembly of powder led us to study the slip casting process further.

**Figure 3:**
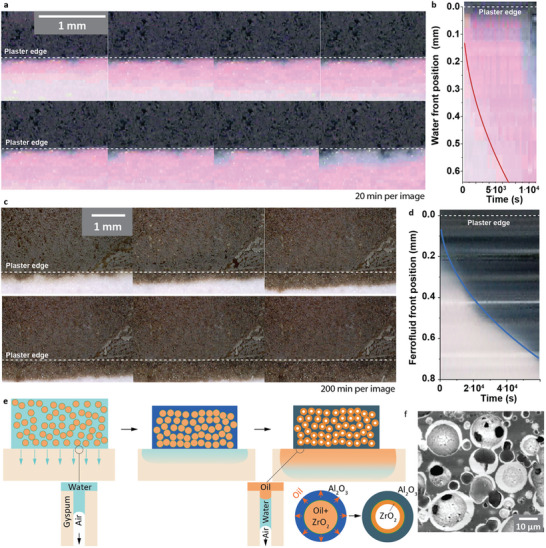
In situ optical characterization of the solvent removal during slip casting of emulsion composites. Time series taken during the casting, a) first 160 min and c) from 160 min to 1360 min. Water contained rhodamine B as dye. Plot of the center slice of the image as function of time superimposed with fit of the position of the solvent front in b) first 80 min with water and d) from 160 to 1360 min with the oil‐based ferrofluid. e) Schematic representation of the two‐step slip casting mechanisms.

The addition of a water‐soluble red dye, Rhodamine B, in the water‐based alumina suspension and the use of black‐colored SPIONs containing oil for the zirconia suspension helps visualize the extraction of the solvents by the gypsum mold over time. The side of the casting setup was covered with a glass slide to allow the direct visualization of the emulsions and gypsum cross‐section using a portable microscope (Figure S8, Supporting Information). Images were recorded over time and analyzed (**Figure** **3**). The extraction of solvent starts as soon as the emulsion is in contact with the gypsum and the liquid extracted by the mold first is the pink‐colored water. The driving force for this solvent extraction is the capillary forces originating from the porous hydrophilic gypsum which lead to the concentration of the ceramic particles close to the mold. This increase in concentration forms a solid casted layer with solvent saturated jammed ceramic particles at the bottom of the emulsion that is growing along the height as the water keeps on getting extracted. This step has been studied in detail for ceramic suspensions and the casted layer thickness increases as $t^{\frac{1}{2}}.$ ^[^ ^36^ ^]^ By extension, it has been established that the depth of the solvent penetration in the gypsum also increases as $t^{\frac{1}{2}}$.

The images taken during casting show that the water is extracted first, with only a few spots where the oil gets in the gypsum that do not spread further (Figure 3a). While gypsum is probably hydro and oleophilic when dry, the water saturated gypsum is preventing the oil from being extracted at this stage.^[^ ^37^ ^]^ Plotting the profile of a single slice taken at the center of the sample as a function of time allows to visualize the penetration depth of the water as function of time (Figure 3b). This confirms that the water depth *d_w_* (*t*) increases as $d_{w}\;\left(t\right)=k_{w}\;\;t^{\frac{1}{2}}$ in the emulsion, with a constant $k_{w}=0.0077\;\mathrm{mm}.s^{-\frac{1}{2}}$, but also shows that the oil starts to penetrate the mould after close to 3 h of casting (10^4^ s, cf. Figure 3b). This sequential behavior has also been observed in emulsion templated porous structure before.^[^ ^26^ ^]^

The same recording is pursued at a slower image acquisition time of 200s to consider the slower oil penetration in the mold (Figure 3c). The images show a similar trend with oil as with water penetration in the mold. The depth of the oil penetration *d_o_* (*t*) follows a similar trend as well, with $d_{o}\;\left(t\right)=k_{o}\;t^{\frac{1}{2}}=0.0029\;t^{\frac{1}{2}}$, however with rate estimated from *k_w_*/*k_o_* 2.6‐fold slower (Figure 3d). This decrease in penetration speed probably originates from the 6‐times higher viscosity of the oil compared with water. These observations allow us to establish a clearer picture of the formation of the emulsion templated composite microstructure.

The water is first removed from the emulsion, leading to the jamming of the alumina particles saturated with water that will lock the oil in place in the droplet. Eventually, the water recesses from the alumina body and so the oil can now be extracted (Figure 3e). We have confirmed that both water and decane can be extracted by a dry porous alumina using contact angle measurements (Figure S3, Supporting Information). Because the oil must go through the alumina first and then the mold or evaporate at the surface, the porous alumina structure around the droplet is now the mold extracting the solvent in this slip casting. This leads to the formation of a thin jammed layer of zirconia forming on all the alumina surface, forming a conformal coating visible on all the microstructures (Figure 3f). Other approaches to remove solvents such as drying in a controlled atmosphere could be used but their effect on the structure will need to be investigated. The thickness of the conformal coating is controlled by φ_*z*_ and we expect that dense droplets could be obtained with an oil phase with a higher solid content. In the case of zirconia dispersed with oleic acid in decane, the rapid increase in viscosity at φ > 0.2 (cf. Figure S9, Supporting Information) preclude the formation of emulsion and thus denser droplets in the final samples. The absence of zirconia particles inside the alumina continuous phase is explained by the similar size of the two populations of particles (cf. Figures S1 and S6, Supporting Information). One would expect the pore size of the porous alumina structure to be smaller than the particles size, assuming that the particles are well dispersed and do not form large flocs during the first slip casting step. This pore size would be too small for the zirconia to migrate in the alumina structure along with the solvent, ensuring the separation.

In summary, the formation of the emulsion templated composite via slip casting is possible and proceed by a two‐step solvent removal, leading to formation of a continuous layers from a hundred nanometers to a few microns thick coating of the pores. Now that the process is better understood, we can start using it to form both porous and dense composites with controlled microstructures.

Starting from the initial emulsion composition developed with water‐based alumina suspension as the continuous phase and zirconia containing oil as the droplet phase, we fabricated porous emulsion‐templated composites. The composition of the water‐based suspension was kept constant, with 20 vol.% alumina dispersed and 7 wt.% of PVA as a function of the water amount while the droplet phase was loaded with an increasing volume fraction of zirconia. The emulsion templated composites are slip cast and heat treated to burn the organic out and sinter the ceramic powder.

**Figure 4:**
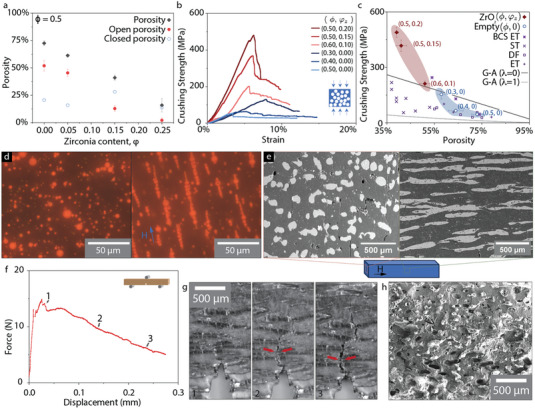
Porous and dense composites with controlled microstructure made by emulsion templating. a) Porosity as a function of the zirconia content at a constant initial volume fraction oil phase ϕ = 0.5. b) Stress‐strain curves in compression of the porous alumina zirconia composite from emulsion templating. c) Crushing strength versus porosity for porous emulsion‐based ceramic and our emulsion templated porous composites. Data obtained from references: BCS Emulsion Templating,^[^ ^21^ ^]^ Sacrificial templating,^[^ ^45^ ^]^ Direct foaming,^[^ ^46^, ^47^ ^]^ Emulsion templating.^[^ ^48^, ^49^ ^]^ The line denoted G‐A corresponds to the prediction from the Gibson‐Ashby model^[^ ^38^ ^]^ for porous alumina with fully closed‐cell λ  =  0 and fully open‐cell structure λ  =  1. d) Optical microscopy images of iron oxides and SPIONS containing oil droplet in water 7 wt.%. PVA solution with and without a static magnetic field applied. e) Microstructure of the alumina‐iron composite made from magnetically templated emulsion after sintering. f) Typical force–displacement curves obtained for the alumina iron templated composites tested in Single Edge Notch Bending. g) Optical images taken during the fracture testing showing the crack propagation. h) SEM of the cross‐section of the composites after testing.

The porosity of the composites decreases almost linearly from 0.75 to 0.20 when the zirconia content in the oil phase increases from φ_*Z*_ =  0 to 0.25, while the oil phase volume fraction is kept constant throughout the whole series at ϕ  =  0.50 (**Figure** **4**a). This linear decrease is expected as more and more of the droplet volume is replaced from oil to zirconia. However, the two‐step slip casting allows for an additional control of the connectivity of the porosity compare with traditional emulsions. The amount of open porosity decreases when the amount of zirconia in the oil phase increases above 5 vol.%, and almost no open porosity is kept at a volume fraction of zirconia of 25%. We explain this behavior with the formation of the shell during the second slip casting step that once it reaches a certain thickness will start closing the connection between touching droplets. This is further illustrated by a close‐up view of the interconnection in Figure S10 (Supporting Information).

The sintered samples are tested in compression. All samples exhibit a graceful failure following the peak load; a behavior typical of brittle foam (Figure 4b). The increase in zirconia content triggers an almost 9‐fold increase in compressive strength, from 54 to 480 MPa and decreases of the porosity from 73% down to 39% for a similar volume fraction of oil in the emulsion. We have two parameters that can now help us control the porosity in our sample, the amount of oil phase ϕ and the solid loading of the oil phase φ_*z*_. Using ϕ  =  0.6 with allowed us to reach lower densities than with ϕ  =  0.5, reaching values close to the empty pores from composition φ_*z*_ =  0. The pore coalescence observed for ϕ  =  0.5 and φ_*z*_ =  0.2 in Figure 2i does not seems to prevent the increase in strength brought by the larger amount of zirconia when compared with the ϕ  =  0.5 and φ  =  0.15. The strengthening effect of zirconia is further highlighted by comparing the crushing strength of the porous ceramic with zirconia shells to the strength predicted by the Gibson‐Ashby model^[^ ^38^ ^]^ for pure alumina (Figure 4c): $\sigma_{c}=\sigma_{f}\;\left(C_{i}\;{\left(\lambda \cdot \rho_{r}\right)}^{\frac{3}{2}}+\left(1-\lambda \right)\rho_{r}\right)$, where *C_i_* =  0.2 for porous brittle materials,^[^ ^38^ ^]^ σ_*c*_ is the crushing strength of the porous materials, σ_*f*_ is the strength of the dense material (taken as σ_*f*_ =  400 *MPa* ^[^ ^20^ ^]^), λ is the fraction of materials present in the cell wall and ρ_*r*_ is the relative density. Whereas the strength of the sample with empty pores falls within the boundary of the model, the strength obtained with the sample with zirconia shells are significantly higher than predicted. This suggests that the material is stronger due to the presence of the zirconia. We decided to compare the structural performance of the alumina‐zirconia porous composites with porous alumina fabricated by other methods but presenting a similar microstructure with spherical pores. The results are summarized in Figure 4c, and the alumina zirconia composites fabricated by emulsion templating present a compressive strength two times higher than alumina porous composites of similar porosity (37% and 39%). The presence of a zirconia shell thus seems to increase the mechanical resistance of the porous composites fabricated through emulsions templating.

Whereas this increase in structural properties could lead to further applications and studies, the possibilities for microstructural control with this technique do not stop here. Using an oil phase containing both SPIONs and iron oxide particles, we can form in situ long metallic fibers in a ceramic matrix using a magnetic field during slip casting. The presence of SPIONs and iron oxide makes the droplets susceptible to a magnetic field. Multiple droplet arrangements can be observed in magnetically responsive droplet systems, from droplet elongation, formation of droplet chains of varying length to finally columnar structures as the intensity of the magnetic field increases.^[^ ^29^, ^39^, ^40^ ^]^ In most cases an increase in magnetic field leads to the formation droplet chains as each droplet form a magnetic dipole under the effect of the macroscopic field. We confirm that this phenomenon occurs with our SPIONs/iron oxide oil phase droplet in a water suspension containing 7 wt.% of PVA (Figure 4d). The presence of a magnetic field from a rare‐Earth magnet in proximity with the emulsion leads to the formation of droplet chains in the direction of the magnetic field.

Emulsion‐templated composites containing alumina in the water phase, SPIONs and iron oxide in the oil phase are then slip cast under a static magnetic field (Figure 1d), then the iron oxide is reduced to metallic iron under reducing gas heat treatment at 600 °C, before being compacted by cold isostatic pressing and finally sintered to almost full density in an inert atmosphere at 1450 °C (see Experimental Section and Figure S11, Supporting Information). Whereas this technique can be used to control the morphology of pores in porous samples as well (see Figure S12, Supporting Information), the pressing step allows us to form fully dense samples. This new composition further exemplifies the role of the particle size in the separation between the droplet and continuous phases. The iron oxide particles, similar in size to the alumina particles, are kept in the droplets, while the SPIONs, two orders of magnitude smaller in size, can be found within the alumina continuous in the reduced green body (see Figure S13, Supporting Information).

The emulsion templated iron/alumina composites present the microstructure we were looking for, with long metallic fiber present within an alumina matrix (Figure 4e). The iron oxide containing droplet chains probably collapse into these dense continuous fibers during the cold pressing step, leading to fiber dimensions of diameter difficult to obtain by other techniques such as 3D printing. The fiber final dimensions can be measured using image analysis (cf. Figure S14, Supporting Information), leading to a median length $l_{50}^{f}=620\;\mu m$ and median diameter $d_{50}^{f}=14\;\mu m$. The diameter of the fibers could be further modulated by changing the droplet size as they originate from the droplet chains formed under the static magnetic field (cf. Figure S13, Supporting Information), whereas the length can be modulated by the field intensity.^[^ ^29^ ^]^

While these microstructures could be interesting for more than enhancing the structural properties depending on the compositions used, we illustrate their usefulness in increasing the fracture resistance of conventionally brittle alumina materials. The presence of metallic fibers in the alumina matrix changes completely the fracture behavior of the composites, as confirmed during a fracture propagation test performed in three‐point bending (Figure 4f). The force–displacement shows an increase in the force after the first crack growth from the notch, with then a slow decrease of the force over a displacement of up to 0.3 mm. The fracture toughness of the composites is $K_{IC}=2.2\pm 0.6\;MPa.\sqrt{m}$, a value that is lower than the value expected for pure alumina (≈3.5 $MPa.\sqrt{m}$ ^[^ ^41^ ^]^). This decrease in initial toughness could be linked with the presence of residual stresses and lower sintering temperature needed for the co‐sintering of iron and alumina.^[^ ^10^, ^11^ ^]^ However, the work of fracture γ_WOF_ of the composites is 385 ± 55 *J*/*m* ^2^, which is an order of magnitude higher than the one of typical alumina.^[^ ^42^ ^]^ Assuming that the increase in toughness is coming from the deformation of the metallic fibers to failure and using the model proposed by Clyne,^[^ ^43^ ^]^ the theoretical work‐of‐fracture of the fiber composites can be estimated by:

(2) $$\gamma_{Wof}^{th}=2\nu_{F}\;s_{f}RW_{d}/\varepsilon_{f}$$

where *s_f_* is the aspect ratio of fiber deformation, i.e., length of fiber elongated beyond the crack plane over the fiber radius, *R* is the radius of fiber, ν_*F*_ =  0.32 the volume fraction of fibers measured by image analysis of the cross section, *W_d_* and ε_*f*_ are work of deformation and failure strain of the fiber respectively. The factor 2 is added compared to the original work to account for the fiber alignment in our composites. For pure iron, the *W_d_* and ε_*f*_ can be estimated to be 45 *MJ* *m* ^−2^ and 0.16 respectively.^[^ ^44^ ^]^ *s_f_* can be estimated from the images of the in situ fracture testing, leading so *s_f_* ≈ 0.42. We finally obtain a $\gamma_{Wof}^{th}\approx 529\;J\;m^{-2}$.

The predicted value is remarkably close to the measured of γ_*wof*_ =  385 ± 55 *J* *m* ^−2^ given the simplicity of the model, showing that the properties of the composite we fabricated through emulsion templating behave as long fiber composites, demonstrating further the interest of this method to make structural materials.

The reasons behind this increase in fracture resistance can be found by looking at the fracture propagation during the test (Figure 4g). The crack can be seen stopping when it encounters a metallic fiber, then deflecting and growing around it, leading to crack bridging by the fibers. The bridging and pull‐out of the fiber is even more apparent when looking at the composites cross‐section post‐fracture (Figure 4h), with evidence of pull‐out and plastic deformation of the metallic fiber protruding from the alumina surface.

In summary, emulsions templating can be used to form complex microstructure in inorganic/inorganic composites that present improved structural properties.

## 3 Conclusion

In conclusion, we demonstrated that by adding particles to both phases in an emulsion we could produce inorganic/inorganic composites with complex microstructures. The control of the rheology of each phase and of the final emulsion through surface active additives is central to the success of the process. We found that PVA act both as a surfactant to stabilize the emulsion droplets and as a gelling agent. The gelling proved to be critical to obtain the desired microstructure in the final composites due to the differences in density of the water and oil phases. The median droplet size we obtain is on average lower than 10 µm but we envision that with different surfactants and higher mixing energies it should be possible to obtain smaller sizes as is the case with conventional emulsions. Using slip casting allows for a controlled solvent removal from the emulsion and using in situ observation we unveiled a two‐step removal process that leads to conformal coating of the inner surface of the droplets by the particles present in the oil phase. The final composites display improved properties directly linked with the microstructural control enabled by the emulsion templating process. The formation of thin zirconia coatings in the internal pores of alumina increases the crushing strengths. Using magnetic field and cold isostatic pressing to form in situ iron fibers within an alumina matrix leads to an increase fracture resistance due to the crack bridging and fiber plastic deformation. We envision that inorganic/inorganic composites fabricated by emulsion templating can open possibilities in the fabrication of energy storage devices, catalytic supportsMicro, or long fiber inorganic composites.

## 4 Experimental Section

### Materials

Al_2_O_3_, alumina powder (SMA6, Baikowski, d_50_ = 0.2 µm), ZrO_2_, zirconia powder (TZ‐3Y‐E, Tosoh), Fe_2_O_3_, iron (III) oxide powder (<5 µm, ≥99%, Sigma–Aldrich), polyvinyl alcohol (PVA, 98–99% hydrolyzed, average M.W. 11000‐31000, Alfa Aesar), Dolapix CA (Zschimmer & Schwarz), oleic acid (Fluorochem Ltd.), decane (≥99%, Sigma‐Aldrich) or oil‐based ferrofluid (EFH‐1, Ferrotec Corp.).

### Water‐Based Slurry Preparation

PVA was dissolved in deionized water at 80 °C to prepare aqueous solutions at 4 and 7 wt.%. Al_2_O_3_ powder was sieved with a 100 µm mesh to break the large agglomerates present in the received powder. Then, the sieved powder was mixed with the PVA solution in a 150 mL HDPE container and 0.5 wt.% Dolapix CA (with respect to weight of powder) was used as dispersant. A planetary centrifugal mixer (THINKY ARE‐250, Thinky Corp.) was used for mixing at 2000 rpm for 2 min, repeated for 3 times, followed by a defoaming step (2200 rpm for 10 min) to get a smooth and homogeneous slurry.

### Oil‐Based Slurry Preparation

ZrO_2_ and Fe_2_O_3_ powder was de‐agglomerated in an ethanol suspension by 6‐h ball milling using a shaker mixer (TURBULA Type T2C, WAB). The ethanol suspension contains 1.9 or 2.0 wt.% to weight of ZrO_2_ and Fe_2_O_3_ powder respectively of oleic acid, 10 vol.% powder and zirconia milling media (ball/powder weight ratio of 3.5/1). By removing the ethanol using a rotary evaporator (Rotavapor R‐300, Buchi Ltd.), oleic acid‐coated powder can be obtained after drying in convection oven at 70 °C overnight. ZrO_2_ or Fe_2_O_3_ powder with precoated oleic acid was weighed and dispersed in decane or oil‐based ferrofluid using Thinky mixer (2000 rpm, 2 min, 3 times).

### Emulsion Preparation and Slip Casting

Next, the emulsification process was carried out using Thinky mixer (2000 rpm, 2 min, 3 times). The emulsion was slip cast in a cylindrical mold and put on a plaster. To prevent the emulsion from sticking to the mold, silicon oil was applied on the walls. It could also help a more uniform shrinkage of the emulsion and reduce cracks formation at the surface or inside the green body. To aid easy release of the green body from the plaster, a piece of filter paper was placed between the emulsion and the plaster, preventing the green body from sticking to the plaster plate and avoiding fracture during removal.

### Magnetically Assisted Slip Casting

To facilitate magnetic templating for emulsions containing a magnetically responsive droplet phase, a static magnetic field was employed during the casting process. The magnetic field was generated by two block neodymium magnets (H x L x T = 50.8 × 50.8 × 25.4 mm; (BH)_max_ = 306 kJ m^−3^; magnetic flux density, B_m_ = 450 mT, Magnet Sales, UK). A 3D‐printed setup was designed to keep the magnets apart, leaving space for a plaster plate for slip casting.

A silicone casting mold was used. It has internal dimensions of 14 × 27 × 20 mm, and wall thickness of 4mm. The mold corners were curved, and its walls were coated with silicon oil to avoid green body breakage due to uneven shrinkage and adhesion with the mold. The emulsion was cast at the center of the plaster for a strong and uniform magnetic field, and the plaster plates were cast to be 45 × 45 × 20 mm, allowing the emulsion to be cast in the center of the magnetic field.

### Drying, Reducing, and Sintering

The green bodies were left to air‐dry at room temperature for at least 96 h. For Al_2_O_3_ – ZrO_2_ composites, a lift furnace was used for sintering in air. Pre‐sintering at 500 °C for 2 h was done for debinding, followed by a 1‐h sintering at 1550 °C, with ramp rates of 2 and 5 °C min^−1^ respectively.

For magnetically assisted slip cast samples with Fe_2_O_3_, the green bodies were first reduced at 600 °C in a 10% H2 / Ar gas for 36 h. Then, cold isostatic pressing (CIP, TCH Instrument Co.) was applied to green bodies at 350 MPa for 5 min to close pores and improve densification. Sintering was carried out in a high‐temperature vacuum furnace with a tungsten heating element and 10% H_2_ / Ar gas at 1450 °C for 1.5 h, with a ramp rate of 5°C min^−1^.

### Microstructural Characterization

Images taken using scanning electron microscope (Auriga, Zeiss, Germany and JSM 6010LA, JEOL, Japan) were used for measuring droplet phase size distribution in image analysis (ImageJ, NIH, USA).

Densities were measured and calculated using Archimedes’ method by employing the theoretical density calculated based on emulsion compositions.

### Rheological Characterization

The Discovery Hybrid Rheometer (DHR‐1, TA Instrument, UK) and a 40‐mm steel parallel plate geometry was used for measuring the rheological behaviors of slurries and emulsions. The testing gap was 500 µm and a solvent trap was used to reduce the effect of evaporation.

Flow ramp tests were carried out with shear rate range from 0.5 to 400 s^−1^ at 20 °C. Oscillation time sweep tests were carried out with fixed oscillation at 1.0 Pa stress, 0.5 Hz frequency for 900 s at 20 °C.

### Time‐Lapse Filming

A digital microscope (Dino‐Lite Premier AM7013MZT) with DinoCapture 2.0 software was used to record the process in time lapse mode once a layer of emulsion was slip casted on plaster. To better distinguish water based continuous phase and oil‐based droplet phase, water soluble Rhodamine B (pink color) was added during preparation of water phase suspension and ferrofluid with brown color was applied as medium of droplet phase. The image sequence recorded by time‐lapse filming was cropped and rearranged as a function of time using Reslice plugin in ImageJ software.^[^ ^50^ ^]^

### Contact Angle Measurements

Raw zirconia powder, iron oxide powder, sieved alumina powder, oleic acid coated zirconia, and oleic acid coated iron oxide were dry pressed into porous powder compacts 13 mm in diameter under a uniaxial pressure of 70MPa. Water and decane contact angles were measured on an OCA 20 machine (DataPhysics Instruments) in a decane/air environment.

### Mechanical Tests

The compressive strength, σ_*c*_ of porous samples was measured by performing compression tests using the Zwick universal test machine (Z010, ZwickRoell) on cubic samples with dimensions of 4.5 × 4.5 × 4.5 mm, with a loading speed of 1 mm·min^−1^. The compressive strength, σ_*c*_ and strain, ε can be calculated using the following equations:

(3) $$\sigma_{c}=\frac{F}{A}$$

(4) $$\varepsilon =\frac{D}{d}$$

where D is displacement, d is sample thickness, A is area of the face under loading and F is applied force.

For three‐point bending test operated on SENB samples, the supporting span of the fixture is 20 mm, while the loading speed is 0.06 mm·min^−1^. The SENB samples were prepared with length of 25 mm, thickness, 2.5 < *d* < 3.0 mm, and width $\frac{d}{2}\;<b<d$. The notch was first cut using a diamond wafering blade (thickness of 0.25 mm), followed by extending the notch using a razor blade and oil‐based diamond suspension (1 µm) for 1 h. The full length of the notched tip, *a_n_*, is prepared to be: $\frac{d}{3}$ < *a_n_* < $\frac{d}{2}$.

A digital camera (Oryx 10GigE, Teledyne FLIR) combined with a telecentric lens (VS‐LTC3.3–45/FS, VS Technology) was applied to record the process at a frame rate of 2 Hz for correction of real displacement during test with a resolution of 1 µm/pixel and imaging field size of 6.46 × 4.85 mm^2^. With image processing using 3D drift correction plugin in ImageJ, image series can be converted into real displacement during SENB test, and contrast adjustment can be applied to track the propagation of crack extension.

The fracture toughness, *K_IC_*, during three‐point SENB test can be calculated using (ASTM‐1820‐11):

(5) $$K_{IC}=\frac{3FL_{s}}{2bd^{\frac{3}{2}}}\;\cdot {\left(\frac{a_{n}}{d}\right)}^{\frac{1}{2}}\times \frac{1.99-\frac{a_{n}}{d}\cdot \left(1-\frac{a_{n}}{d}\right)\cdot \left[2.15-3.93\frac{a_{n}}{d}+2.7{\left(\frac{a_{n}}{d}\right)}^{2}\right]}{\left(1+\frac{2a_{n}}{d}\right)\cdot {\left(1-\frac{a_{n}}{d}\right)}^{\frac{3}{2}}}$$

F is the applied force at which F – d curve diverges from a linear relation.

The total energy of fracture process *W_f_* can be calculated from the force‐displacement curve, hence work of fracture, γ_WOF_, can be calculated from:

(6) $$W_{f}=\int Fd\delta \;$$

(7) $$\gamma_{WOF}=\frac{W_{f}}{2A_{f}}\;$$

where *d*δ is change of displacement and *A_f_* =  *b*(*d* − *a_n_*) is area of fractured surface.

## Conflict of Interest

The authors declare no conflict of interest.

## Supporting information



Supporting Information

## Data Availability

The data that support the findings of this study are available within this article and its supplementary materials.
