# Supplementary material for: Inorganic/Inorganic Composites Through Emulsion Templating
Source: Adv Mater. 2024 Dec 20;37(7):2411352. doi: 10.1002/adma.202411352 (PMC11837884; doi:10.1002/adma.202411352)
Supplement: Supplementary file 1 — Supporting Information [file ADMA-37-2411352-s001.docx]

**Inorganic/inorganic composites through emulsion templating**

Tianhui Jiang^1^, Shitong Zhou^1^, Yinglun Hong^1^, Erik Poloni^1^, Eduardo Saiz^1^, Florian Bouville^1^

^1^Centre for Advanced Structural Ceramics, Department of Materials, Imperial College London, London (UK)

**Figure S1**. Size distribution of sieved alumina particles dispersed in 7 wt.% PVA water solution with 0.5 wt.% Dolapix CA as dispersant, assessed by laser diffraction

**Figure S2**. Size distribution of zirconia particles in decane (precoated with 1.9 wt.% oleic acid), measured by dynamic light scattering.


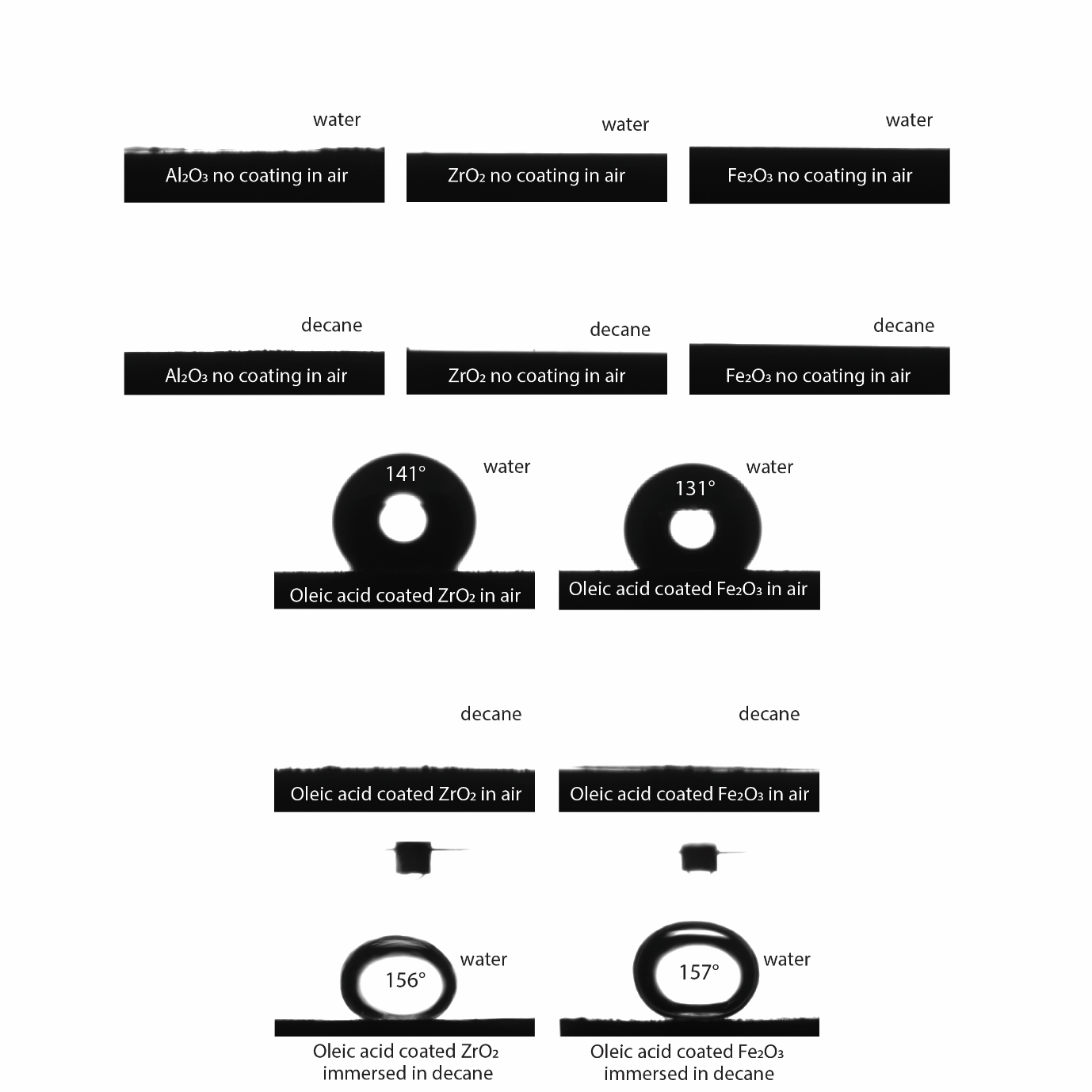


**Figure S3**. Contact angle measurement with porous ceramic compact as substrate and either decane or water droplet of controlled volume added in top.

**Table S1**. Average power law fitting results of viscosity – shear rate (η-$\dot{\gamma}$) plots in range of $\dot{\gamma}$ = 100-300 s^-1^ for three groups of emulsions ($\varphi_{Z}$ = 0, 0.1 and 0.2) with increasing volume fraction of decane phase ($\phi$ = 0.1-0.6). Fitting was taken on plots of three trials of experiments for each sample separately to obtain mean values and percentage errors.

| φ_Z_ | φ | n | | K | | R^2^ |
| --- | --- | --- | --- | --- | --- | --- |
| 0.2 | 0.1 | 0.78 | ±6.9% | 0.26 | ±30.6% | 0.9898 |
| 0.2 | 0.2 | 0.73 | ±0.4% | 0.51 | ±3.8% | 0.9989 |
| 0.2 | 0.3 | 0.71 | ±2.1% | 0.77 | ±9.5% | 0.9973 |
| 0.2 | 0.4 | 0.72 | ±4.3% | 0.97 | ±18.6% | 0.9993 |
| 0.2 | 0.5 | 0.60 | ±1.9% | 3.15 | ±9.5% | 0.9998 |
| 0.1 | 0.2 | 0.77 | ±2.9% | 0.45 | ±15.3% | 0.9975 |
| 0.1 | 0.3 | 0.74 | ±2.3% | 0.60 | ±7.1% | 0.9970 |
| 0.1 | 0.5 | 0.68 | ±1.8% | 1.82 | ±6.4% | 0.9996 |
| 0.1 | 0.6 | 0.50 | ±0.3% | 6.35 | ±2.5% | 0.9996 |
| 0.0 | 0.1 | 0.75 | ±2.2% | 0.39 | ±9.6% | 0.9942 |
| 0.0 | 0.3 | 0.74 | ±4.2% | 0.66 | ±23.0% | 0.9914 |
| 0.0 | 0.4 | 0.71 | ±2.2% | 1.10 | ±8.5% | 0.9961 |
| 0.0 | 0.5 | 0.74 | ±1.0% | 1.33 | ±6.1% | 0.9987 |
| 0.0 | 0.6 | 0.66 | ±0.4% | 2.82 | ±2.8% | 0.9961 |

| a | b |
| --- | --- |
|  | 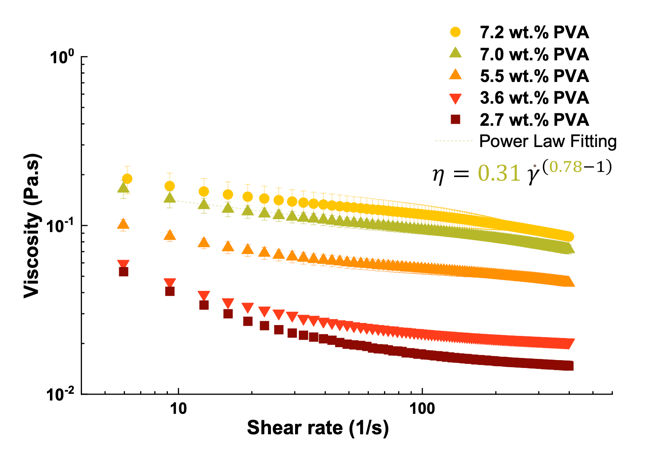 |

**Figure S4**. **a**) plot of power law exponent against droplet phase volume fraction, $\phi$, for droplet with different zirconia content ($\varphi_{Z}$ = 0, 0.1, 0.2), based on fitting results with power law model in Table S1. B. **b**) Flow ramp plots of 20 vol.% Al2O3 – 0.5 wt.% Dolapix CA – water suspension with PVA concentration increasing with respect to its water solution, the shear rate range was 1 – 400 s-1. Power law fitting (dashed line and the equation containing fitting results) is shown for 7.0 wt.% PVA curve.


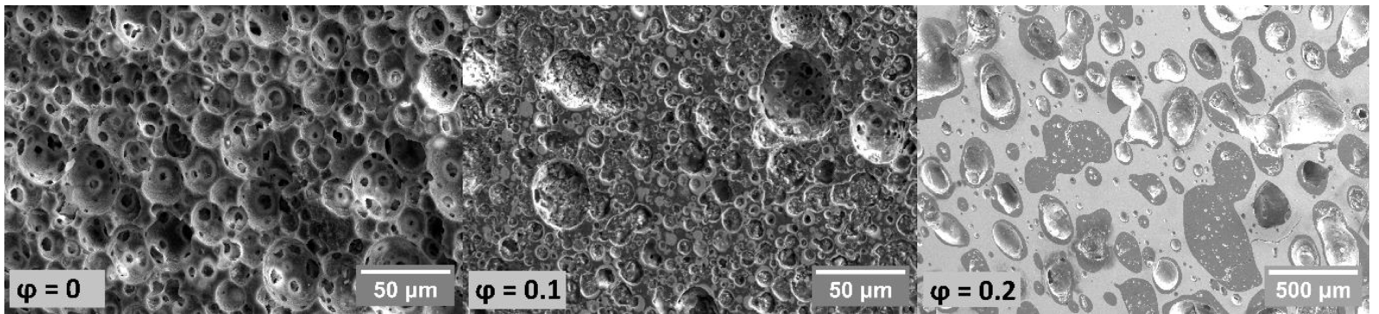


**Figure S5**. SEM images for $\phi= 0.6$ with increasing zirconia content $\varphi_{z}$ from 0 to 0.2.

**Figure S6**. Size distribution (by volume) of iron (III) oxide particles dispersed in decane (precoated with 2.0 wt.% oleic acid) ), measured by dynamic light scattering.


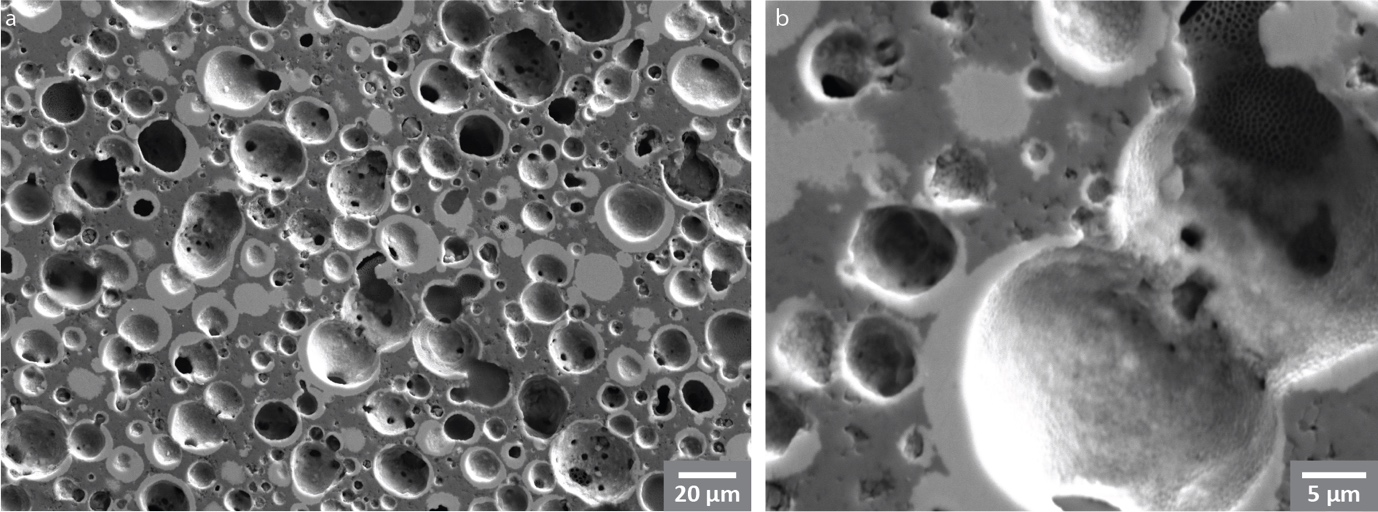


**Figure S7**. **a**) SEM images of alumina zirconia emulsion composites $\phi=0.50$ and $\varphi_{z}=0.15$. **b**) Close up view from **a** of two zirconia-loaded oil droplets partially coalesced.


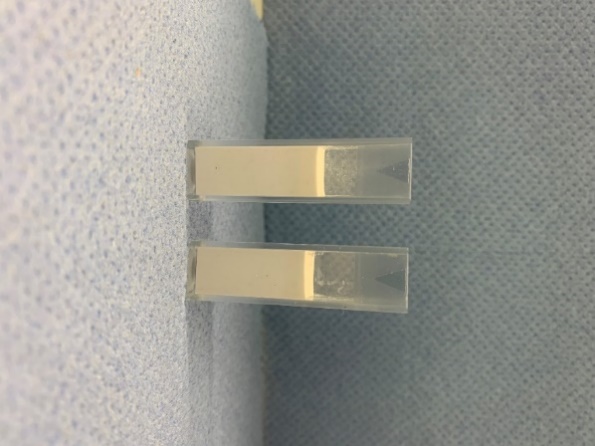

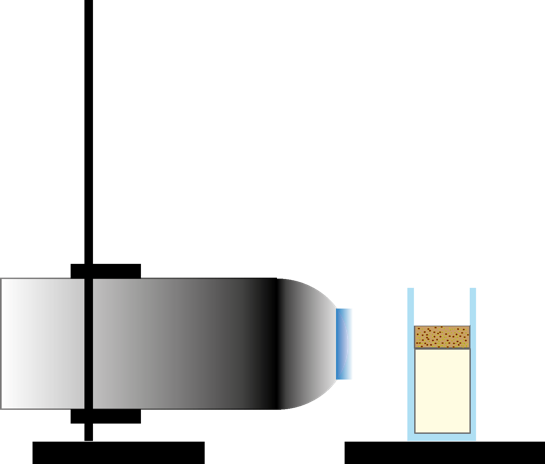

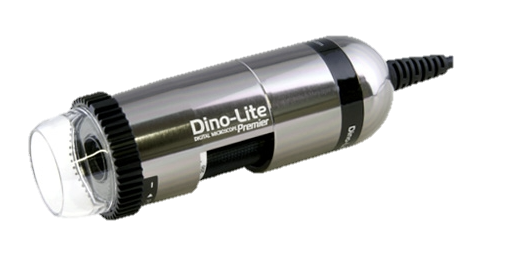


**Figure S8**. Graphic illustration of setup used for time-lapse filming to record emulsion solidified on plaster.


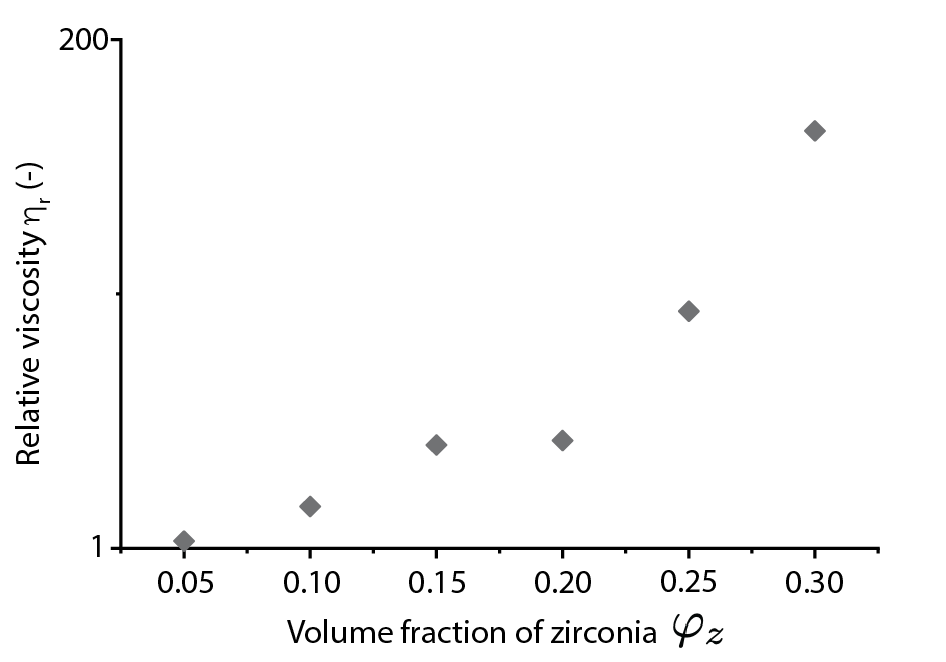


**Figure S9**. Evolution of the relative viscosity $\eta_{r}$ of decane zirconia suspension against ZrO2 volume fraction. The relative viscosity $\eta_{r}$ is defined as the viscosity of the suspension at high shear rate divided by the viscosity of decane.


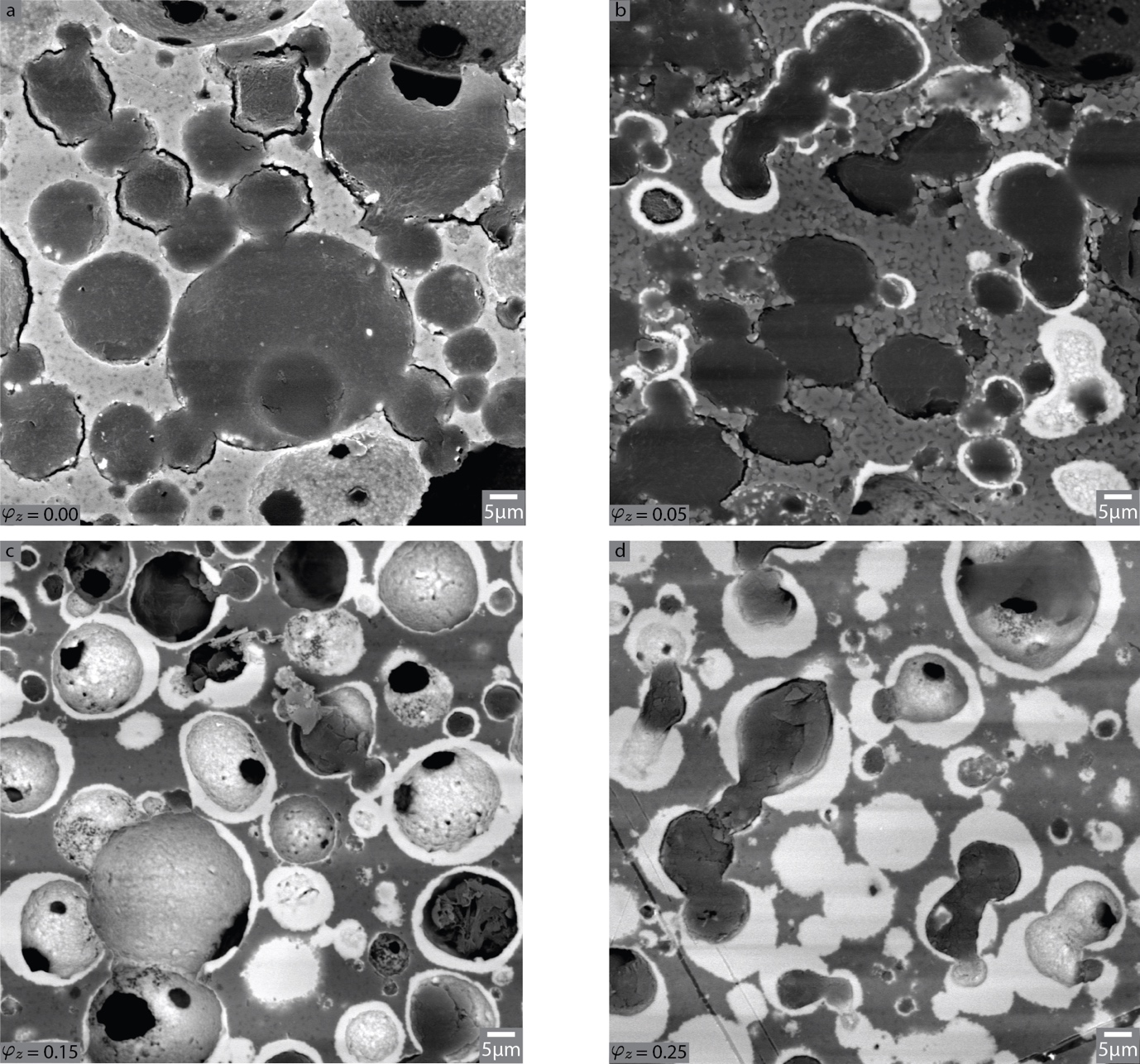


**Figure S10**. SEM images of the pore’s interconnection with different volume fraction of zirconia a $\varphi_{z}=0$, b $\varphi_{z}=0.05$, c $\varphi_{z}=0.15$ and d $\varphi_{z}=0.25$at a constant $\phi=0.5$

**Figure S11**. XRD pattern of sample F-30-F-30 measured on green body, reduced sample (36-hr), and sintered sample in powder form


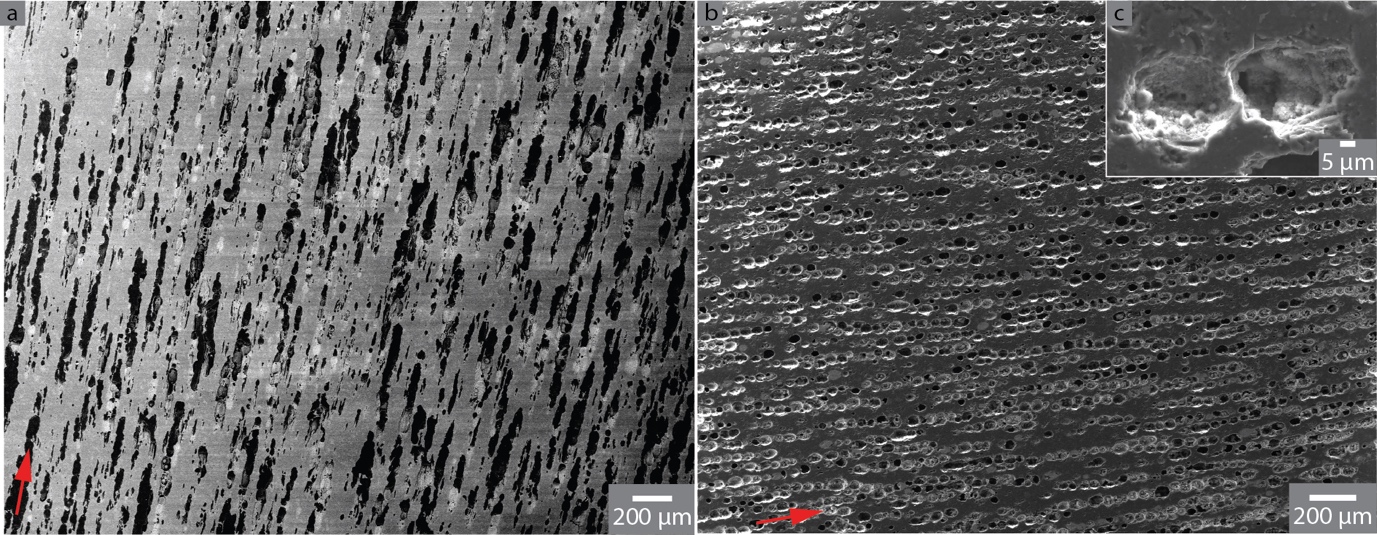


**Figure S12**. Sem images of porous ceramic with droplet chain like porosity fabricated using SPIONs loaded oil phase **a** (ϕ$=0.10, \varphi=0)$, and with a metallic iron coating after sintering an emulsion composite with $\phi=0.20$ and iron oxide in the oil phase $\varphi_{F}=0.20$ in a reducing atmosphere in **b**. Insert **c** showing the decoration of the pores by the iron. All samples were slip cast under a static magnetic field with direction represented by the red arrow on the SEM.


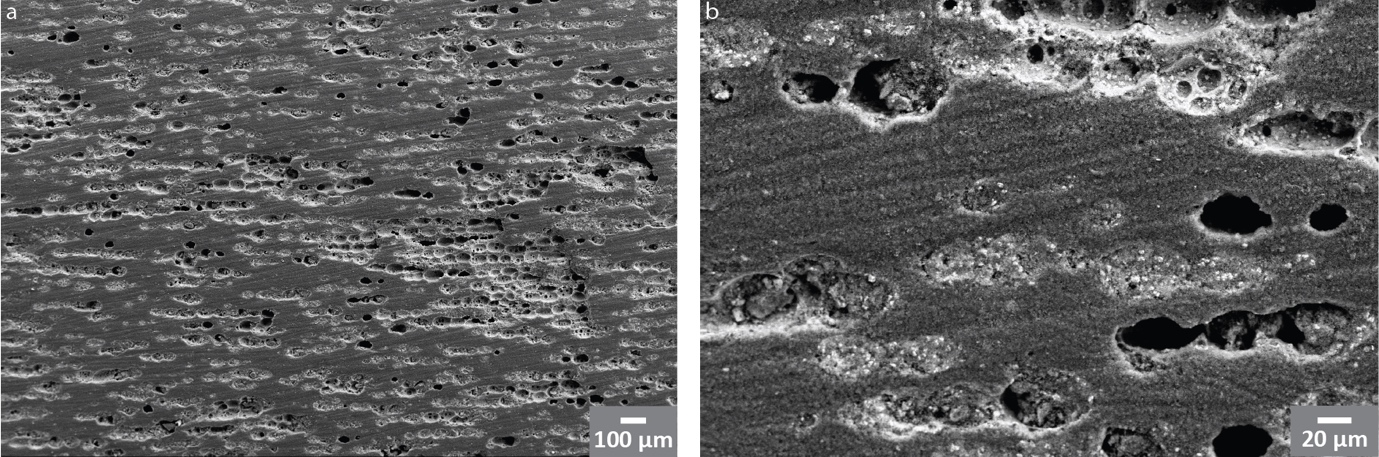


**Figure S13**. **a**) SEM images of alumina/iron oxide emulsion composites $\phi=0.20$ and $\varphi_{F}=0.30$ after reduction and partial sintering. **b**) Close up view from **a** showing the iron in the droplets and SPIONs in the droplets and continuous phase.


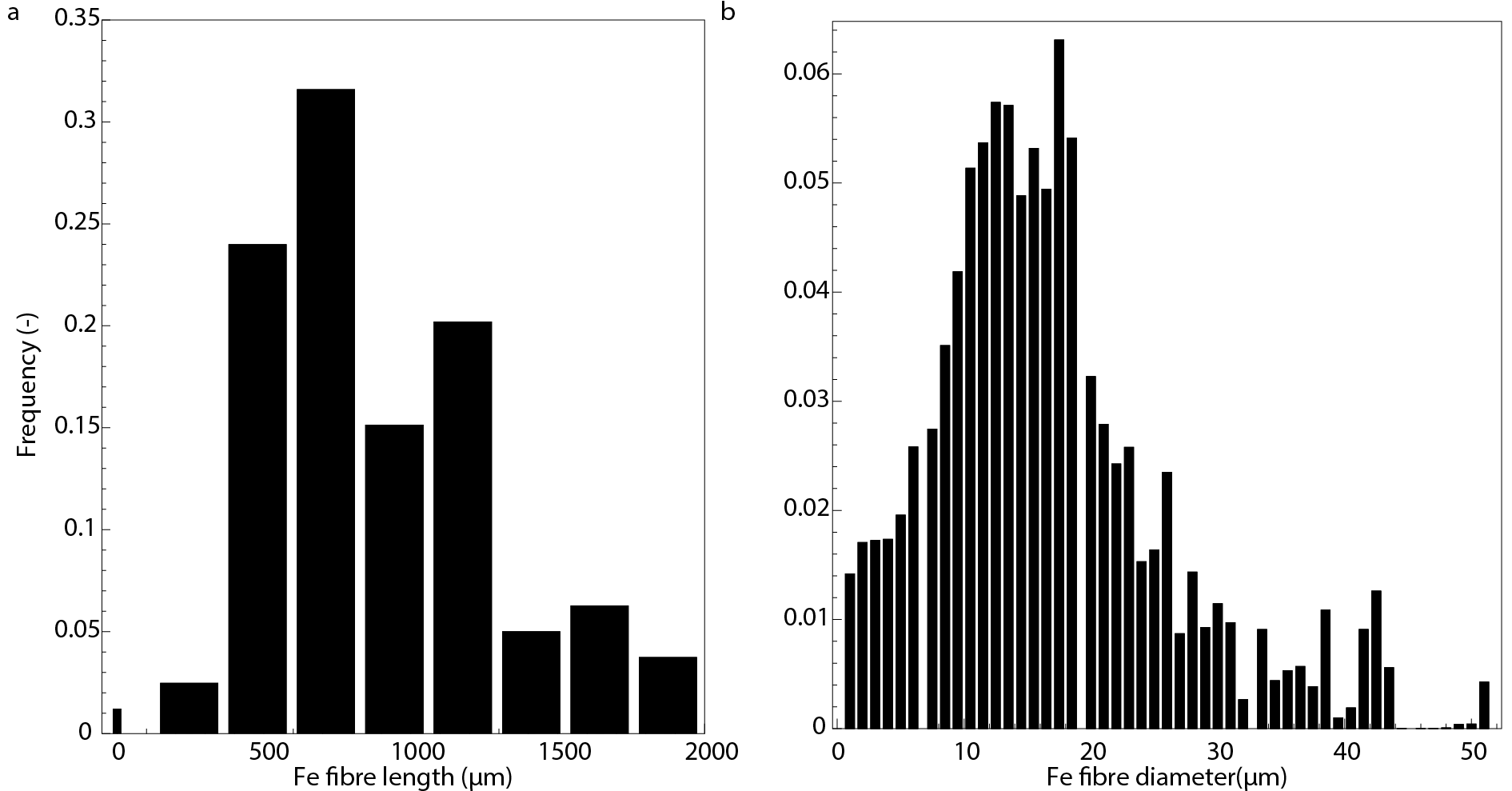


**Figure S14**. Distribution of a length and b diameter of the metallic iron fibre alumina composites measured by image analysis
